# Use of pyrolytic carbon coated beads (Durasphere) to treat glottic failure: an experimental study in dogs

**DOI:** 10.1016/S1808-8694(15)30540-1

**Published:** 2015-10-19

**Authors:** Domingos Hiroshi Tsuji, Flavio Akira Sakae, Rui Imamura, Luis Fernando Ferraz, Luiz Ubirajara Sennes

**Affiliations:** 1 Associate Professor of ENT - University of São Paulo (USP). Head of the Larynx Group - University of São Paulo Medical School; 2 PhD in Sciences - Department of ENT - USP. Assistant ENT - PUC, Campinas; 3 PhD in Sciences - Department of ENT - USP. Assistant ENT - University Hospital - USP; 4 Graduate Student - Department of Pathology - USP. Assistant Physician - Department of Pathology - USP; 5 Associate Professor of ENT - USP

**Keywords:** vocal cords, larynx, speech

## Abstract

There is no ideal tissue or substance to be injected in the vocal folds. The objective of the present study was to assess the use of Durasphere in canine vocal fold injection.

**Materials and Methods:**

in six adult dogs we injected 0.3 mL of Durasphere in the middle third of the thyroarytenoid muscle and 0.3 mL of saline solution in the contralateral vocal fold. The animals were slaughtered after seven days (three dogs) and after 90 days (three dogs). We analyzed the inflammatory process in the vocal fold and in the lamina propria of the vocal folds.

**Results:**

in the vocal muscle which received Durasphere there was a significantly more intense inflammation when compared to the control muscle - there was a moderate lymphomodular infiltrate after seven days and mild after 90 days. We did not observe foreign bodies nor granulomas. On the lamina propria there was a mild inflammatory process in the two groups, without difference between them.

**Conclusion:**

this is a substance of proven biocompatibility in humans, with preliminary and unprecedented results and its injection in canine vocal folds caused a moderate inflammatory process after seven days and mild after 90 days, without foreign bodies or granuloma formation.

## INTRODUCTION

Normal laryngeal functioning requires the vocal folds to come close to each other for phonation and protection of the lower airways. When this reunion fails because of paralysis, atrophy or vocal fold fibrosis, glottal insufficiency ensues^1^, ^2^, ^3^. Patients unable to properly close their glottis can suffer of weak and blowy voice and chronic aspiration^3^.

Vocal fold injection is the technique used to correct this problem. The most commonly used substances to push the vocal folds medially are: Teflon, gelfoam, autogenous fat, collagen and autologous fascia^1^, ^4^, ^5^.

There is no ideal tissue universally accepted to inject in the vocal fold, and each material has its pros and cons. One new substance, Durasphere (Carbon Medical Technologies, St. Paul, Minnesota), made of pyrolithic carbon particles, suspended in aqueous gel^6^ was approved by the FDA (Food and Drug Administration) to treat urinary incontinence. Its function is to promote a closure (bulging) when injected in the submucosa of the urethra near the bladder neck, thus reestablishing bladder competence.

The ideal injectable substance must be biocompatible, durable, non-migratory, little immunogenic, of easy injection and which does not alter the viscoelastic properties of the vocal fold^7^. Considering that Durasphere has potential advantages which can be ideal for vocal fold injection, the goal of the present study was to assess the use of Durasphere as a substance to inject in canine vocal folds to treat glottic insufficiency.

## MATERIALS AND METHODS

This study was approved by the Ethics Committee for Research Project Analysis - CAPPesq of the Clinical Board of the University of São Paulo Medical School Hospital (protocol # 353/04).

In this study we used adult dogs, of both genders, from the research animal's center of the Medical School of the University of São Paulo, without a defined race and mean weight of 10 kg.

**Figure 1:**
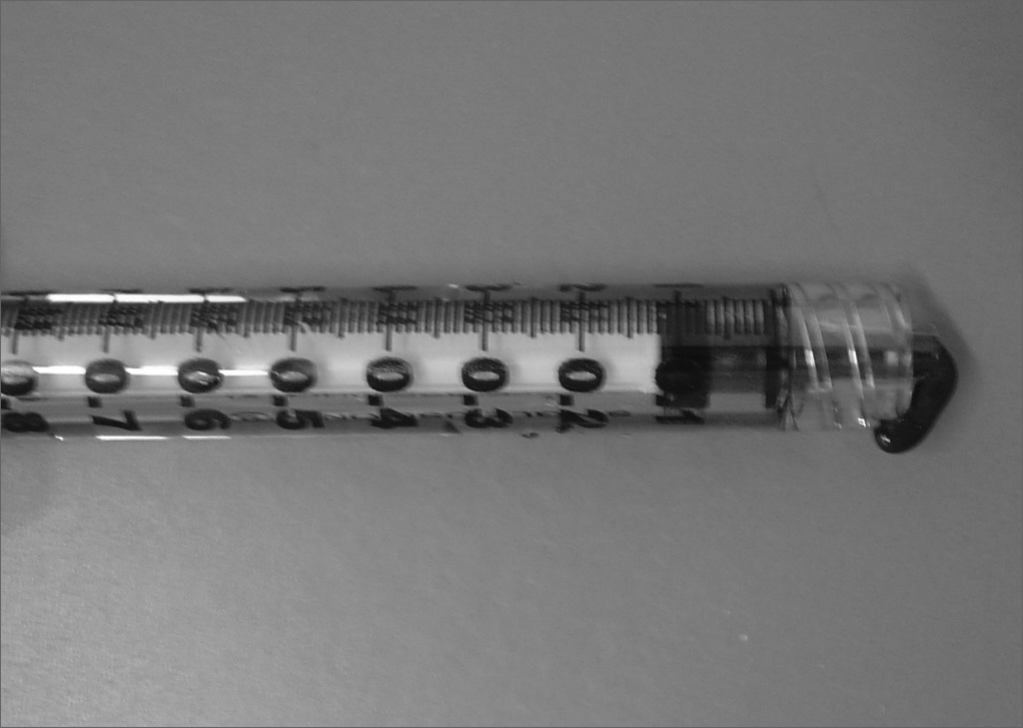
Durasphere in a 1mL syringe.

Durasphere (Carbon Medical Technologies, St. Paul, Minnesota) is made of globules coated by pyrolithic carbon suspended in aqueous gel at 2.8% glucan (Figure 1). The particles have sizes which vary between 251 and 300μm.

With the animal in supine position and previously sedated, we carried out a suspension laryngoscopy to expose the animal's glottis. Under light microscopy we injected 0.3mL of Durasphere in the middle third of the right vocal fold, lateral to the vocal process, at about 3mm of depth in the thyroarytenoid muscle, using a 19 gauge needle. The left vocal fold was used as control, and was injected with 0.3mL of 0.9% saline solution in the same site compared to the other fold.

The animals were slaughtered after 7 days (3 dogs) and 90 days (3 dogs) and submitted to total laryngectomy with dissection of all the structures adjacent to the larynx. We separated the two vocal folds and obtained a five millimeters thick fragment from the middle third of the intermembranous portion of each vocal fold, involving epithelium, lamina propria and vocal muscle. Durasphere - appeared as a well outlined mass of pasty consistency - was carefully removed from the vocal fold in order to facilitate the paraffin bloc cutting, because the results from the prior pilot study with five dogs showed that after paraffin impregnation, Durasphere became very hard, making it difficult to cut the paraffin with the microtome, and this caused muscle tissue laceration, impairing histological evaluation.

The vocal folds were fixed in 10% formaldehyde, dehydrated in 95% ethylic alcohol, cleared with xylol, impregnated by paraffin melted in a 60 degree oven and after that they were cut with the microtome at a 5um thickness.

The slides were dyed with hematoxylin eosin. We analyzed the quantity and type of local inflammatory infiltrate in the vocal muscle and the lamina propria of both vocal folds, for that we used a quantitative and qualitative method.

The inflammatory process was qualitatively graded in mild, moderate or severe, based in the infiltrate intensity. We also noticed foreign body reaction and granuloma formation.

**Figure 2:**
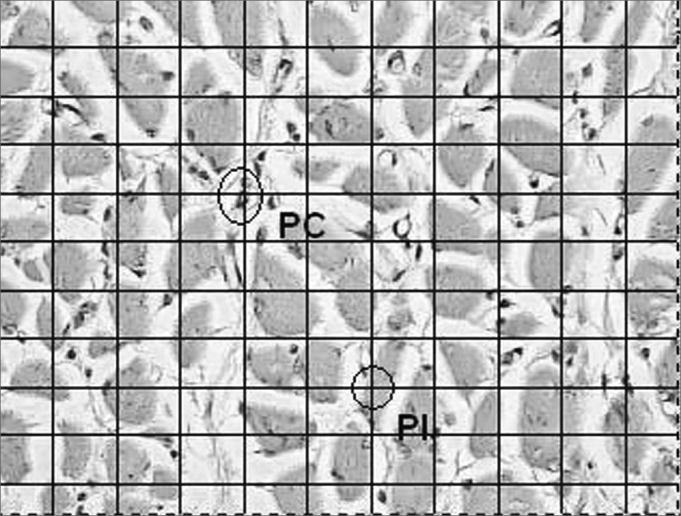
Coronal histological cross-section of the canine right vocal fold from dog slaughtered after 90 days. Dye: hematoxylin-eosin. Quantitative analysis of the inflammatory process. Squared grid with 10 vertical and 10 horizontal lines, making up a total of 100 points of intersection. The points were counted in two groups: coinciding points (PC) which had inflammatory cells and inclusion points (IP) corresponding to the remaining points. The inflammatory process of each region was measured by means of an average value of the coinciding points of the 10 fields analyzed (400x).

For the quantitative analysis in order to assess the quantity of the inflammatory reaction we chose 10 visual fields at random from the vocal muscle slide and 10 of the lamina propria with optical magnification of 400 times, on top of which we placed a squared grid with 10 vertical and 10 horizontal lines, making up a total of 100 points of intersection. The points were counted in two groups: coinciding points which had inflammatory cells and inclusion points corresponding to the rest of the points. The inflammatory process of each region was measured through an average of the coinciding points of the 10 fields analyzed (Figure 2). We compared the inflammation percentages between the right (Durasphere) and left (control) vocal folds, and we grouped the dogs from the two groups (7 days and 90 days), because of the small sample of our study. We used the Wilcoxon non-parametric test for paired samples, with a 5% significance level.

## RESULTS

We did not notice intraoperative or late complications in the animals of this study. In two animals there was a mild extrusion of the substance at the time of injection in the vocal muscle.

In the macroscopic inspection of the larynx we observed a bulging in the vocal fold with Durasphere after 7 days and after 90 days. We did not see Durasphere in other laryngeal regions.

**Figure 3:**
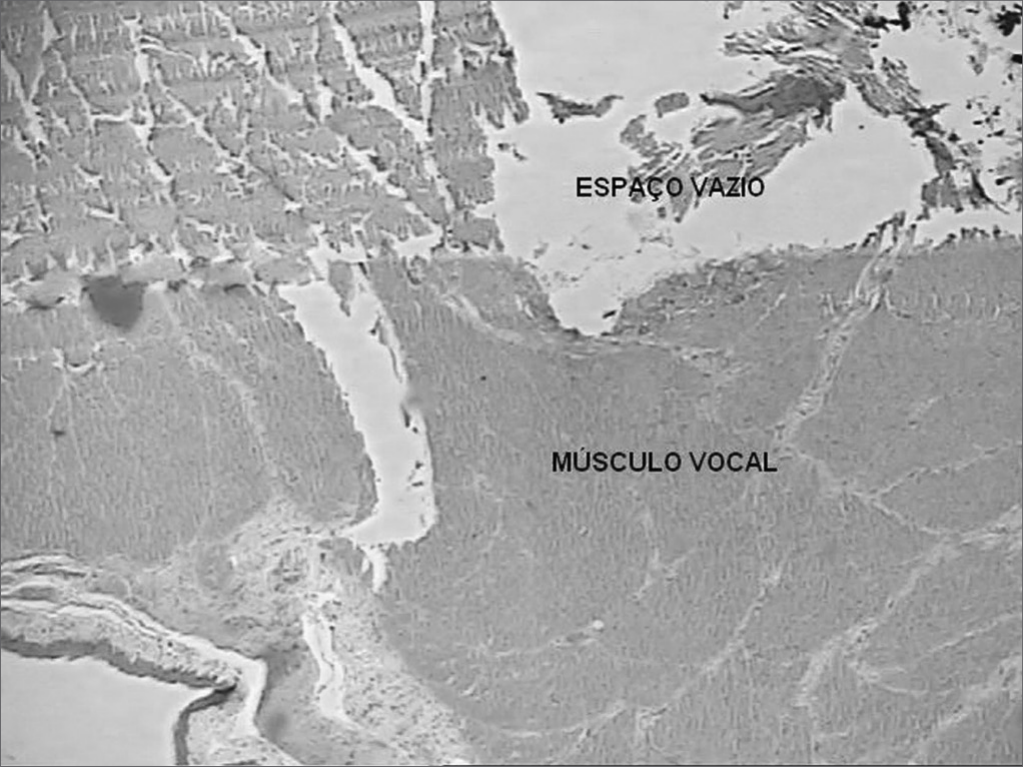
Coronal histological cross-section of the right vocal fold of a dog slaughtered after 90 days. Dye: hematoxylin-eosin. We notice an empty space in the vocal fold after removal of the Durasphere (50x).

**Figure 4:**
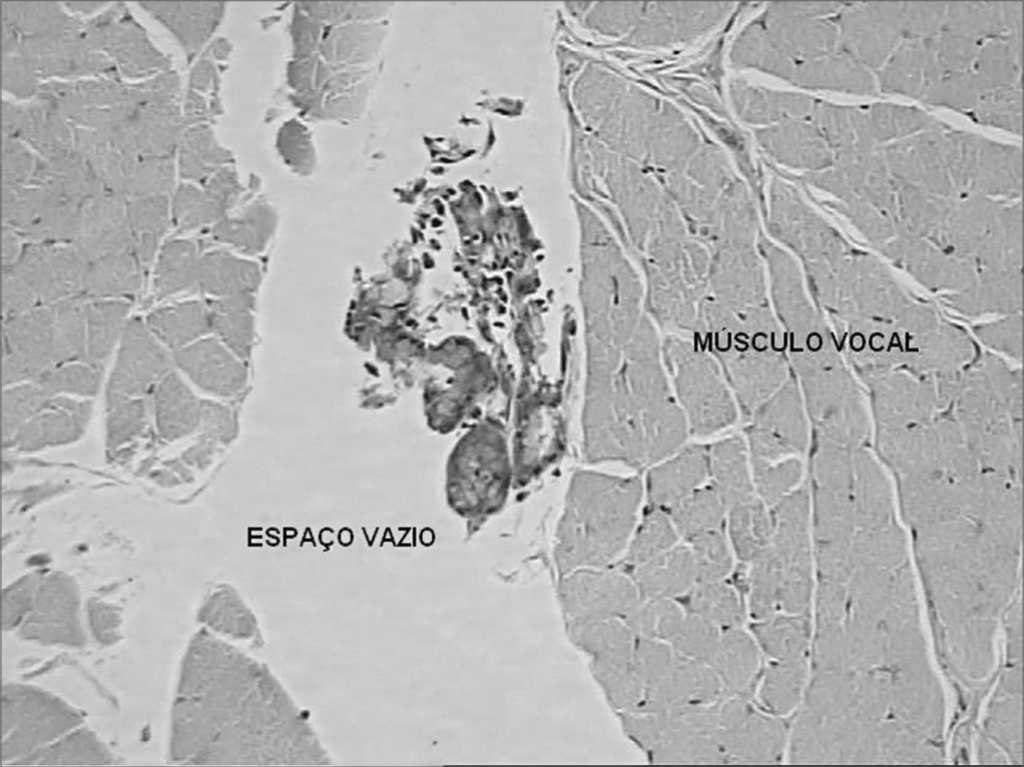
Coronal histological cross-section of the right vocal fold (Durasphere) from a dog slaughtered after 90 days. Dye: hematoxylin-eosin. We notice a mild lymphomononuclear infiltrate in the vocal muscle adjacent to the empty space (200x).

In the histologic analysis of the slides we noticed that the space previously occupied by Durasphere was empty (Figure 3). There was moderate lymphomononuclear infiltrate adjacent to this space in the vocal muscle within 7 days and a mild infiltrate after 90 days (Figure 4). There were no foreign bodies or granulomas.

**Figure 5:**
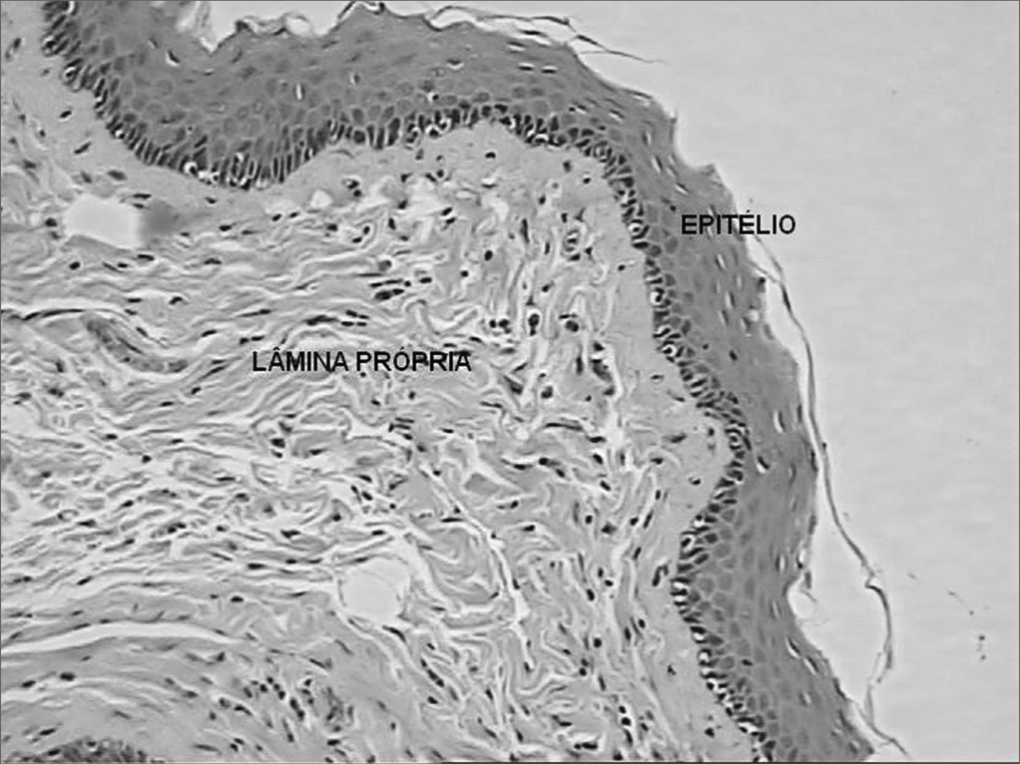
Coronal histological cross-section of the left vocal fold (control) from a dog slaughtered after 90 days. Dye: hematoxylin-eosin. We notice the lamina propria with mild lymphomononuclear infiltrate (200x magnification).

In the lamina propria of the vocal folds with Durasphere and the control ones we noticed a mild lymphomononuclear infiltrate with 7 days and 90 days (Figure 5).

The quantitative study of the inflammatory process revealed that in the vocal fold with Durasphere the inflammation was significantly greater than that in the control muscle. In the lamina propria there was no difference regarding the inflammatory process between the two vocal folds (Chart 1).

**Chart 1:** Quantitative analysis of the inflammatory process in 6 dogs (p <0.05)

% Inflammation	RVF (Durasphere) N = 6	LVF (control) N = 6	p
Lamina propria	7,0 ± 2,4	5,8 ± 3,0	0,6
Muscle	9,5 ± 8,4	0,9 ± 0,8	0,046

## DISCUSSION

Durasphere has been clinically used in the submucosa of tissues (urethra or anal sphincter); however, its use in the larynx to treat glottic insufficiency has never been attempted, and this motivated this initial study in dogs.

Durasphere injected in the vocal muscle of dogs showed a moderate inflammatory reaction, one significantly greater than that in the control muscle, and such result was expected since it is a synthetic substance. Synthetic particles like teflon^8^ can cause an intense inflammatory process; however, Durasphere in the vocal muscle did not cause an intense inflammatory process, foreign body reaction or granulomas.

Durasphere was initially tested as injection substance in the periurethral tissue of dogs in order to establish its biocompatibility and migration potential. In assessments held up to 2 years there was mild to moderate inflammatory process from 7 to 28 days, which evolved to chronic mild inflammatory process in the periods of 3, 6, 12 and 24 months, involving the build up of macrophages^9^.

Human biocompatibility has been proven in clinical practice. Lightner et al.^6^ did a multicentric, randomized, controlled and double-blinded study with Durasphere and bovine collagen as injection substances for the treatment of stress urinary incontinence in 355 women between 26 and 84 years. Results during the 1 year follow up revealed that Durasphere is a safe substance and an effective alternative for the treatment of urinary incontinence, which was in agreement with other studies^10^, ^11^. Durasphere effectiveness has also been proven in the treatment of fecal incontinence^12^.

Synthetic substances can migrate to other tissues.

Prior studies^8^, ^13^ suggest that particles larger than 50μm have a lower likelihood of migration. Although our study did not assess a possible lymphatic migration when injected in the vocal fold, an experimental study in dogs^9^ showed that when injected in the periurethral tissue there was no migration to the lymphatic tissue. We believe that the same happens when it is injected in the vocal fold. The low migration risk can be due to the fact that Durasphere is made up of larger particles (251 and 300μm).

Resorption is also a problem associated with the injection of substances. This study did not assess objectively a possible Durasphere resorption; however, the evaluation after 3 months showed the presence of Durasphere in all the dogs and since it is a synthetic substance, the likelihood of resorption is lower. A study^7^ with hydroxyapatite reveals the possible need for reinjections because of the absorption of the aqueous gel which carries the particles, this may also happen with Durasphere.

In our study, Durasphere was injected in the vocal muscle and despite the substance being made up of larger particles (251 to 300μm), there was no resistance to its injection, probably because the quantity injected was small, 0.3mL, while in the urethra, where the injection is in the submucosa, the quantity is much higher, 4.8mL in average^6^. We inject it in the vocal fold because it does not alter its viscoelastic properties, keeping the histologic characteristics of the lamina propria intact, added to the fact that there was no significant inflammatory process in the lamina propria when compared to the control vocal fold.

## CONCLUSION

This is a substance of proven biocompatibility in humans, with preliminary and unprecedented results from its injection in canine vocal folds, which caused a moderate inflammatory process after 7 days and mild one after 90 days, without foreign body or granuloma reactions.

## References

[1] Duke SG, Salmon J, Blalock D, Postma GN (2001). Fascia Augmentation of the Vocal Fold: Graft Yield in the Canine and Preliminary Clinical Experience. Laryngoscope..

[2] Hsiung MW, Woo P, Minasian A, Mojica JS (2000). Fat Augmentation for Glottic Insufficiency. Laryngoscope..

[3] Ford CN, Bless DM (1986). Clinical Experience with Injectable Collagen for Vocal Fold Augmentation. Laryngoscope..

[4] Imamura R, Sennes LU, Chung D, Bohadana S, Tsuji DH (2003). Injeção de gordura na prega vocal: efeitos do local de injeção sobre a configuração glótica e a distribuição espacial da gordura injetada. Rev Bras Otorrinolaringol..

[5] Zaretsky LS, Shindom L, Detar M, Rice DH (1995). Autologous Fat Injection for Vocal Fold Paralysis: Long-Term Histologic Evaluation. Ann Otol Rhinol Larygol..

[6] Lightner D, Calvosa C, Andersen R, Klimberg I, Brito CG, Snyder J (2001). A New Injectable Bulking Agent for Treatment of Stress Urinary Incontinence: Results of a Multicenter, Randomized, Controlled, Double-blind Study of Durasphere. Urology..

[7] Chhetri DK, Jahan-Parwar B, Bhuta SM, Hart SD, Berke GS (2004). Injection Laryngoplasty with Calcium Hydroxylapatite Gel Implant in an in Vivo Canine Model. Ann Otol Rhinol Laryngol..

[8] Boedts D, Roels H, Kluyskens P (1996). Laryngeal Tissue Responses to Teflon. Arch Otolaryngol..

[9] Carbon Medical Technologies. Durasphere for the Treatment of Gastroesophageal Reflux: A Post-Market Study. Investigation Plan, 2004.

[10] Anderson RC (2002). Long Term Follow-Up Comparison of Durasphere and Contigen in the Treatment of Stress Urinary Incontinence. Journal of Lower Genital Tract Disease..

[11] Madjar S, Covington-Nichols C, Secrest CL (2003). New Periurethral Bulking Agent for Stress Urinary Incontinence: Modified Technique and Early Results. J Urol..

[12] Davis K, Kumar D, Poloniecki J (2003). Preliminary Evaluation of an Injectable Anal Sphincter Bulking Agent (Durasphere) in the Management of Faecal Incontinence. Aliment Pharmacol Ther..

[13] Malizia AA, Reiman HM, Myers RP (1984). Migration and Granulomatous Reaction After Periurethral in Polycef (Teflon). JAMA..

